# Quality of life of dental patients treated with laser surgery: A scoping review

**DOI:** 10.1002/hsr2.1368

**Published:** 2023-06-21

**Authors:** Dhanushka Leuke Bandara, Kehinde K. Kanmodi, Afeez A. Salami, Jimoh Amzat, Ruwan D. Jayasinghe

**Affiliations:** ^1^ Department of Oral Medicine and Periodontology University of Peradeniya Peradeniya Sri Lanka; ^2^ School of Health and Life Sciences Teesside University Middlesbrough UK; ^3^ Faculty of Dentistry University of Puthisastra Phnom Penh Cambodia; ^4^ Cephas Health Research Initiative Inc Ibadan Nigeria; ^5^ Department of Oral and Maxillofacial Surgery University College Hospital Ibadan Nigeria; ^6^ Department of Sociology Usmanu Danfodiyo University Sokoto Nigeria; ^7^ Department of Sociology University of Johannesburg Johannesburg South Africa

**Keywords:** dental patients, laser, laser surgery, quality of life

## Abstract

**Background and Aims:**

The use of lasers has been increasing in various surgical procedures. Its specific characteristics have conquered the scalpel used to a major extent in certain surgical procedures. This scoping review aimed to assess the empirical evidence that exists on the quality of life (QoL) of dental patients treated with laser surgery.

**Methods:**

This scoping review was conducted in accordance with the Arksey and O'Malley's guidelines for scoping reviews. Four electronic databases (PubMed, SCOPUS, CINAHL Complete, and APA PsycInfo) were systematically searched through a stepwise approach, informed by the PEO (Population [P], Exposure [E], and Outcome [O]) framework, to retrieve literatures relevant to the review question. After a two‐staged and Rayyan‐aided screening process, only those literatures meeting the inclusion criteria were included into the review. From the included literatures, data were extracted, collated, summarized, and presented.

**Results:**

The literature search retrieved 246 articles, of which only 10 articles were selected according to the inclusion criteria. Five of the studies were from the United Kingdom and three were from Italy. Study designs were either cohort (60%) or randomized controlled trials (40%). A vast variation was observed in the study populations. The used QoL instruments were mostly disease/condition‐specific and oral cancer was the most reported disease in the included articles. The patients who underwent laser surgery had better QoL on the 7th day postoperatively, although it was not significant in later days.

**Conclusion:**

Depending on the indication, Laser is a safe surgical approach that could enhance the clinical outcome as well as the QoL of dental patients. Laser effects were more significant in the domain of postoperative pain. Due to the limited number of studies evaluated in this review, further longitudinal studies are needed to corroborate the findings of this review.

## 1 INTRODUCTION

### 1.1 Background

Since its introduction in the year 1960, research has continued on the use of LASER (Light Amplification by the Stimulated Emission of Radiation) in dentistry. In surgical dental procedures, lasers have been used in a wide range of procedures such as gingivectomy, frenectomy, incisional or excisional biopsies, lesion removal and in making incisions for flap access.^1^ Based on the wavelength and other key characteristics, the main types that have been used for surgical procedures are carbon dioxide (CO_2_), neodymium–yttrium–aluminum–garnet (Nd:YAG), and erbium–yttrium–aluminum–garnet (Er:YAG) lasers and the diode laser.^1^

The recent rapid advances in laser technology as well as the expanding knowledge of bio‐interactions of various laser systems have broadened the clinical application of lasers in dentistry.^2^ Evidence demonstrates enhanced clinical results in laser‐assisted surgical procedures with reduced morbidity due to ablation, vaporization, hemostasis, and field sterilization leading to several benefits including minimum bleeding, reduced swelling, less discomfort, and shorter healing times.^3^ However, whether these benefits affect the quality of life (QoL) of an individual compared to the other surgical techniques in dentistry is not widely discussed.

### 1.2 QoL

The World Health Organization (WHO) defines QoL as “an individual's view of their place in life concerning their objectives, aspirations, standards, and concerns in the context of the society in which they live.”^4^ Thus, the standard indicators of the QoL include physical health, psychological condition, amount of autonomy, social interactions, beliefs, and relationship to important environmental elements.

Similarly, within the field of healthcare, health‐related quality of life (HRQOL) is a multidimensional concept that reflects the impact of health status, the treatment's efficacy, and other elements impacting people's lives.^5^ Thus, it is usually assessed via various indicators of self‐perceived health status as well as physical and emotional well‐being.

### 1.3 Instruments to assess the QoL

Evaluating the QoL is a difficult task that requires a variety of measurements to reflect subjectivity and multidimensionality. Thus, the available instruments are developed based on empirical considerations to assess different domains of the QoL and are mainly classified as generic or condition/disease‐specific.^6^

Generic instruments target numerous areas of QoL across a variety of patient or illness groups, whereas condition/disease‐specific instruments measure the health parameters that are essential to a specific group of patients.^7^ When assessing the QoL, the use of both generic and condition/disease‐specific measures would be more beneficial as the general measures may be used to compare QOL across health conditions, while condition/disease‐specific measures directly target the health condition and seem to be more clinically relevant.

The most widely used generic QoL instruments in the literature are Medical Outcomes Study Short‐Form 36 (MOS SF‐36), World Health Organization Quality of Life Assessment (WHOQOL), Short‐Form Health Survey (SF‐12), and Visual Analog Scale (EQ‐VAS).^8^ Oral Health Impact Profile (OHIP‐14) and the University of Washington Quality of Life Questionnaire (UW‐QOL) are examples of specific instruments. The main domains assessed in these instruments are mostly based on physical health, psychological health, social relationships, and the immediate environment. For example, the UW‐QOL is a self‐administered questionnaire designed specifically for head and neck cancer patients to assess health‐related QoL.^9^ Following its introduction in the year 1993, the UW‐QOL questionnaire has improved with five versions and the latest version; 4.1 assesses 14 domains including intimacy and fears of recurrence. All versions from version 1 to 4.1 commonly assessed nine domains which included pain, appearance, activity, recreation, swallowing, chewing, speech, and functional limitations. In version 3, two new domains; taste and saliva were added while the employment domain was dropped. In the version 4, mood and anxiety were included to assess the emotional domain. Each component is scored from 0 (lowest) to 100 (best) where the combined score will be calculated by averaging all scores. The three global questions which were included from the version 2 of UW‐QOL covers HRQOL before the cancer diagnosis, during the past 7 days and the overall QoL during the past 7 days.^9^ In contrast, the OHIP‐14 assesses the impact of oral health problems on an individual's life via seven domains; functional limitation, physical pain, psychological discomfort, physical disability, psychological disability, social disability, and handicap.

Although there is a lack of a conceptual model for the assessment of QoL, it has become a key outcome measure in health‐related fields and the systematic review by Pequeno et al. also has shown that the QoL instruments could help make informed decisions about disease management.^10^ Furthermore, as QoL is often considered to be an expected health outcome, there are trial models proposed combining the clinical outcomes with the health‐related QoL.^11^

Thus, this scoping review mainly aimed at finding out the existing literature on the QoL of dental patients treated with laser surgery.

## 2 METHODS

### 2.1 Review design

The design of this study was based on the stepwise procedure for conducting scoping reviews; developed by Arksey and O'Malley which includes identification of the research question, identification of relevant literature, literature selection, data extraction and analysis, collation, summarization, and presentation of results.^12^

### 2.2 Research question

This study intends to address the principal research question: What empirical evidence exists on the QoL of dental patients treated with laser surgery?

### 2.3 Search strategy

The PEO (Population [P], Exposure [E], and Outcome [O]) framework formed the search strategy adopted in this scoping review.^13^ The population of interest was dental patients, the exposure was laser dental surgery, and the outcome was QoL.

The scoping search was performed on January 21, 2023, to retrieve relevant literature from the PubMed, SCOPUS, CINAHL Complete, and APA PsycInfo databases. This search was conducted using the terms “laser,” “dental surgery,” “oral surgery,” “maxillofacial surgery,” “periodontal surgery,” “quality of life,” and “wellbeing,” and with the aid of Boolean operators (“AND” and “OR”). Tables A1, A2, A3 show the search strings used for the search from each of the selected databases.

### 2.4 Deduplication

The citations of the literature obtained from the database search were exported into the Rayyan software for deduplication. Following deduplication, based on the selection criteria, the retrieved literature was screened for inclusion in the review.

### 2.5 Eligibility criteria

A set of criteria was adopted for the exclusion from or inclusion of literature in this scoping review. The inclusion criteria were: (a) empirical research articles, of any design, published in peer‐reviewed journals; (b) articles published in English language; (c) articles reporting the QoL of dental patients treated with laser dental surgery; and (d) articles with accessible full text.

The exclusion criteria were: (a) review articles, editorials, letters, commentaries, and any other nonempirical research article types published in peer‐reviewed journals; (b) articles published in French, Spanish, Italian, or any other non‐English language; (c) articles reporting the QoL of orthognathic surgery patients, nondental patients treated with laser surgery, and other dental patients not treated with nonlaser surgery; and (d) articles without accessible full text.

### 2.6 Screening and selection

The screening process was two‐staged, aided by the Rayyan software. In the first stage, two reviewers screened all papers independently by title and abstract while the second stage involved full‐text screening. Disagreements among the reviewers were discussed and clarified. However, on occasions where disagreements were not amendable, a third reviewer was invited to make the final decision. Only the literature that met the inclusion criteria was included in the scoping review.

### 2.7 Data extraction and analysis

From the yielded literature, the following data were extracted; citation details (names of authors, article title, and the year of publication), country of origin, study type, aims of the study, study population size and characteristics, Instrument tools used for the QoL measurement, type of the laser used and key findings relevant to the scoping review question. Extracted data were analyzed through narrative synthesis. Meta‐analysis was not done in this study due to heterogeneity of data.

## 3 RESULTS

### 3.1 Search results

**Figure 1:**
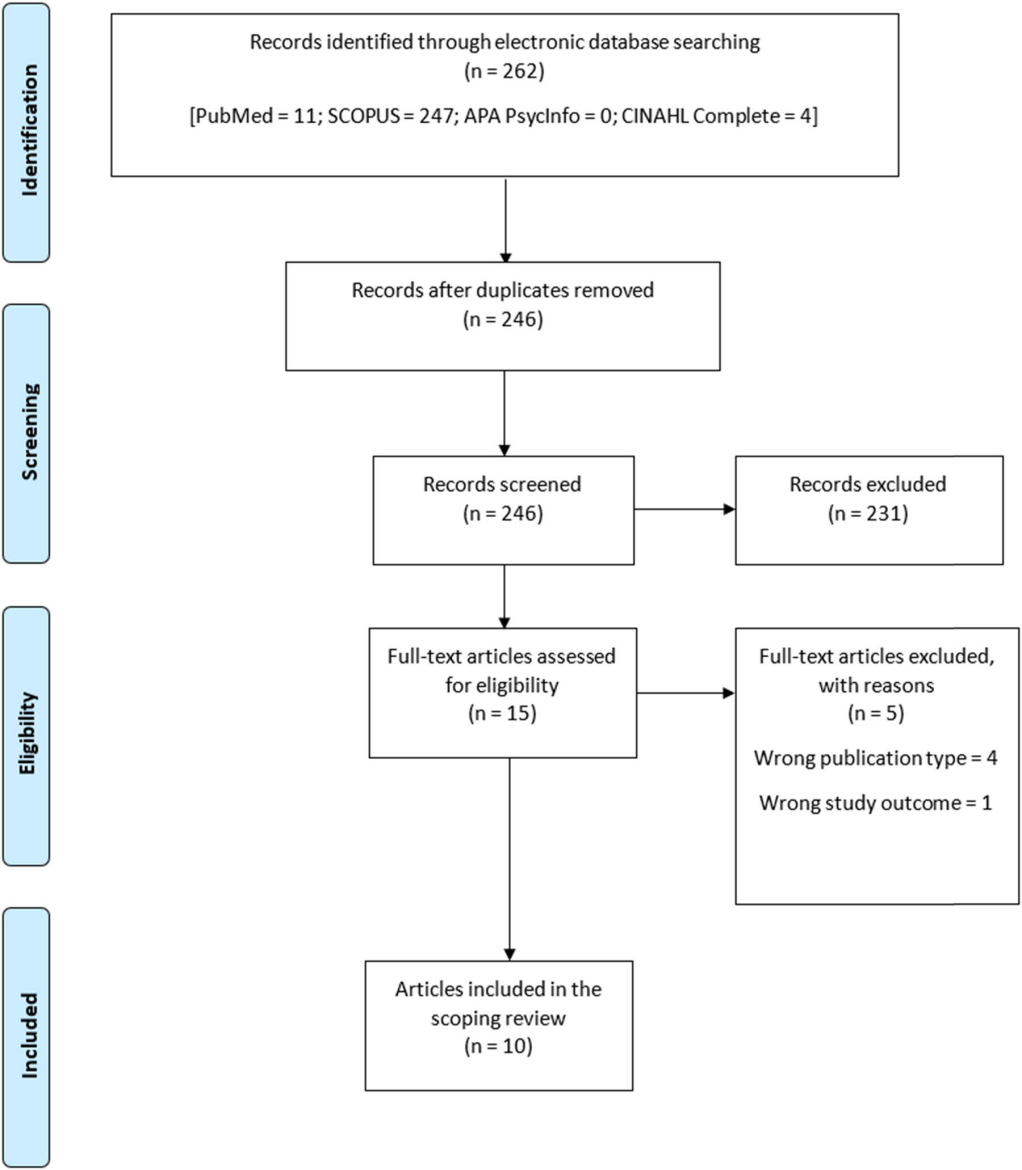
Flowchart of the article selection process.

A total of 262 publications (PubMed = 11, SCOPUS = 247, APA PsycINFO = 0, and CINAHL Complete = 4) were retrieved from the database search. Of these 262 publications, 16 were found to be duplicates and were removed. The remaining 246 publications were screened and only 10 articles were found relevant for inclusion in the review. Figure 1 illustrates the article selection process in accordance with the PRISMA diagram.

### 3.2 Characteristics of the included studies

Of the included studies, 90% were from Europe that had been conducted in three different European countries (Table 1). Five of the 10 studies were conducted in the United Kingdom, three in Italy, and one each in the United States^15^ and Turkey.^17^

**Table 1:** Sources of the included literature by country.

No.	Author(s)	Year of publication	Country	Continent
1	Gardenal et al.^14^	2022	Italy	Europe
2	Rosenthal et al.^15^	2021	USA	North America
3	Breeze et al.^16^	2018	United Kingdom	Europe
4	Ozcelik et al.^17^	2016	Turkey	Europe
5	Broccoletti et al.^18^	2015	Italy	Europe
6	Giovannacci et al.^19^	2015	Italy	Europe
7	Zuydam et al.^20^	2005	United Kingdom	Europe
8	Rogers et al.^21^	2002	United Kingdom	Europe
9	Rogers et al.^22^	2001	United Kingdom	Europe
10	Rogers et al.^23^	1999	United Kingdom	Europe

According to the study designs, the majority were prospective cohort designs or randomized controlled designs (Table 2). All studies were mostly related to the adult population.

**Table 2:** Summary of the included literature.

No.	Author(s)	Study design	Aims of the study	Target population	Laser type	Intervention	Instrument/measurement tool	Measured Parameter	QoL domains assessed	Duration of the intervention	Key findings related to QoL
1	Gardenal et al.^14^	Retrospective cohort study	To assess postoperative outcomes including postoperative pain and quality of life (QoL)	Patients with oro‐pharyngeal cancers (*n* = 81, Mean age of 69 ± 13 years)	CO_2_ waveguide (WG) laser	Laser resection through three different surgical access	UW‐QOL version 4	HRQOL	Twelve domains— pain, appearance, activity, recreation, swallowing, chewing, speech, shoulder, taste, saliva, mood, anxiety	One month postoperatively	The domains of Saliva, Taste, Pain and Speech demostrated highest scores. Chewing and Shoulder complaints had the lowest scores. Mean composite QoL score was 77 ± 14. Very low postoperative pain is reported with fast recovery ensuring good overall QoL.
2	Rosenthal et al.^15^	Randomized controlled trial	To compare postoperative pain and QoL	Patients with carcinoma, dysplastic/benign lesions, premalignant/early stage oral cancer. (*n* = 62)	Flexible fiber CO_2_ laser	Excision of the lesion using flexible fiber‐optic CO_2_ laser (*n* = 32) and electro‐cautery (EC) (*n* = 30)	UW‐QOL version 4 and the Performance Status Scale for head and neck cancer (PSS‐HN).	HRQOL	UW‐QOL version 4: 12 domains—pain, appearance, activity, recreation, swallowing, chewing, speech, shoulder, taste, saliva, mood, anxiety	1st, 3rd, 7th, 14th, 21st, and 28th postoperative days	QoL was lowest at 7th POD and in both groups returned toward baseline QoL by 28th POD. Better UW‐QOL scores and PSS scores, quicker return to normal diet and faster return to work was observed in the laser treated group. However, the results were not statistically significant.
								Performance status scale	PSS‐HN: three main domains—normalcy of diet, eating in public and speech		
3	Breeze et al.^16^	Prospective cohort study	To determine patient‐reported QoL following treatment for oral cancer	Patients treated for oral cancer (*n* = 102, mean age = 65.5 years)	Not defined	Excision via conventional method and using Laser	UW‐QOL version 4	HRQOL	Twelve domains—pain, appearance, activity, recreation, swallowing, chewing, speech, shoulder, taste, saliva, mood, anxiety	Twelve months postoperatively	Laser excision resulted better QoL compared with the other excision techniques. The postoperative pain scores in the laser group were almost half those in the nonlaser group. Improved QoL was observed in patients underwent laser resection of early anterior tongue cancer.
4	Ozcelik et al.^17^	Randomized controlled trial	To evaluate the effects of the use of diode laser for graft harvesting and palatal wound irradiation on postoperative morbidity and root coverage outcomes	Patients with isolated recession defects (*n* = 52)	Diode laser	Root coverage with coronally advanced flap (CAF)+ de‐ epithelialized gingival grafts (DGG). Deepithelization was done via; a scalpel blade extra‐orally (Control group) and using a diode laser (test group)	Turkish versions of OHIP‐14.	OHQoL	Seven domains— physical pain, psychological discomfort, physical disability, psychological disability, social disability, handicap, functional limitations	Seventh day and 6 months postoperatively	The overall scores demonstrated statistically significant differences between the groups. Test group reported significantly decreased postoperative morbidity associated with palatal donor‐site surgery and better postoperative QOL scores.
5	Broccoletti et al.^18^	Randomized Controlled trial	To estimate the effects of excision method, on the early postoperative sequelae of nondysplastic oral lesion removal	Patients with nondysplastic oral lesions (*n* = 344, mean age = 61.7 years)	Er:YAG laser	Removal of the lesions using Er:YAG laser and the traditional scalpel	Italian version of OHIP‐14	OHQoL	Seven domains—physical pain, psychological discomfort, physical disability, psychological disability, social disability, handicap, functional limitations	Seventh day postoperatively	The laser appeared to be faster and less painful than traditional scalpel
							QoL	QoL	Five domains—work efficiency, social activities, appetite, depression and anxiety		
6	Giovannacci et al.^19^	Randomized control trial	To compare pain, HRQOL and need for painkillers during the postoperative course	Patients with similar surgical interventions (*n* = 163, mean age = 58 years)	Nd:YAG Laser	Oral soft tissue surgery performed with Nd:YAG laser (*n* = 77), quantic molecular resonance (QMR) scalpel (*n* = 45) and cold blade (*n* = 41).	Italian translation of the modified version of HRQOL questionnaire proposed by Majid.^24^	HRQOL	Five domains—social isolation and working isolation, eating ability and diet variation, speaking ability, sleep impairment, physical appearance. Each domain carried several questions to rate four‐point likert scale (0=Never, 4=Very much).	Seventh day postoperatively	Laser group had better, statistically significant improvement and lower postoperative pain than cases performed with cold blade. This may be possibly associated with bio‐modulating effect of the laser.
7	Zuydam et al.^20^	Prospective cohort study	To examine the association between the speech and swallowing aspect of HRQOL and selected clinical parameters, and particularly to determine those that are predictive of good outcomes at 1 year after surgery.	Patients undergoing surgery for squamous cell carcinoma (*n* = 278, mean age = 62 years)	Not defined	Resection of the lesion	UW‐QOL version 4	HRQOL	Twelve domains— pain, appearance, activity, recreation, swallowing, chewing, speech, shoulder, taste, saliva, mood, anxiety	More than 12 months postoperatively with a median of 39 months	Primary surgical closure/laser surgery were the main predictors of good swallowing and in good speech
8	Rogers et al.^21^	Prospective cohort study	To explore the relationship between an 11‐point clinical examination and HRQ OL.	Patients with oral and oropharyngeal squamous cell carcinoma (*n* = 130, mean age = 60 years)	Not defined	Laser resection (*n* = 19) and flap surgery (*n* = 98).	UW‐QOL version 2	HRQ OL	Nine domains—pain, disfigurement, activity, recreation, swallowing, chewing, speech, shoulder function, and employment	Twelve months postoperatively	Changes in the UW‐QoL cumulative scores at the 1% level only depended on the type of operation (The difference between pre‐op vs. post‐op in Laser group was less).
9	Rogers et al.^22^	Prospective cohort study	To identify which factors were associated with prolonged length of stay (LOS) following surgery	Patients undergoing surgery for oral and oropharyngeal squamous cell carcinoma (*n* = 130, mean age = 61 years)	Not defined	Surgery included primary closure, laser excisions or split skin grafts	UW‐QOL version 2	HRQOL	Nine domains—pain, disfigurement, activity, recreation, swallowing, chewing, speech, shoulder function, and employment	Twelve months postoperatively	Patients who underwent Laser had better physical functioning, less hospital stay and better QoL compared with the Free flap surgery patients. The laser group stayed a median of 2 days at the hospital compared with 16 days following microvascular free flap reconstruction.
							European Organization for Research and Treatment of Cancer (EORTC) C30				
									Six function scales—physical functioning, role functioning, cognitive functioning, emotional functioning, social functioning and global health status/QoL. There are nine additional symptom items including fatigue, appetite loss, dyspnea, sleep disturbance, and financial impact.		
10	Rogers et al.^23^	Prospective cohort study	To investigate the disease‐specific functional status\	Patients with oral cancer treated (*n* = 48)	CO_2_ laser	Surgical resection. T1 lesions were done by laser/primary closure	UW‐QOL version 1	HRQOL	The nine domains—pain, disfigurement, activity, recreation, swallowing, chewing, speech, shoulder function, and employment.	Twelve months postoperatively	Patients underwent laser treatment had higher cumulative QOL score at 1 year Dropped in QoL at 3 months was mainly due to pain, recreation, and chewing domains.

Abbreviations: Er:YAG, erbium–yttrium–aluminum–garnet; HRQOL, health‐related quality of life; Nd:YAG, neodymium–yttrium–aluminum–garnet; QoL, quality of life; UW‐QOL, University of Washington Quality of Life Questionnaire.

### 3.3 Assessment outcomes of QoL in patients that underwent laser‐assisted surgeries

The surgical procedures that have been considered were involved with the removal of oral/oro‐pharyngeal carcinoma,^14^, ^16^, ^20^, ^21^, ^22^, ^23^ precancerous/early cancer^15^/nondysplastic lesions,^18^ oral soft‐tissue surgeries,^19^ and root coverage procedures.^17^ Although four studies had not defined the type of laser used, other studies reported the outcomes with CO_2_ laser,^14^, ^15^, ^23^ Nd:YAG laser,^19^ Er:YAG laser,^18^ and the diode laser.^17^ Within the 10 papers reviewed, among the various QoL assessment tools utilized for the evaluations, the most commonly used instrument was version 4 of the UW‐QOL.^14^, ^15^, ^16^, ^20^

The main domains that have been assessed in all studies were pain, physical appearance, physical ability, and oral functions such as chewing and speech. Different studies have evaluated the outcomes at different time intervals. However, most of the studies have assessed the outcome on the 7th day postoperatively and the ratings have been defined according to the instrument used (see Table 2). All studies had shown a positive QoL outcome with the use of lasers and the most significant improvement was noted in postoperative pain.

## 4 DISCUSSION

### 4.1 Summary of the findings

Following the development of the various types of lasers and their characteristics, the use of lasers has increased while the evidence on how it affects the QoL is often not adequately reported. The strict eligibility criteria used in the selection process retrieved only 10 articles out of the initial 246 articles. The selected studies for this scoping review demonstrated diverse characteristics with a variety of target groups, duration of the evaluations, different research designs, and different types of QoL instruments.

In literature, the terms HRQOL and QoL frequently appear interchangeably. However, it is crucial to record and explicitly specify whether overall QoL or HRQOL was assessed.^25^ Among the different methods to assess QoL/HRQOL, self‐administered questionnaires completed by the patient are now considered to be the most practical form of assessment.^26^ The majority of the included studies measured HRQOL while only a few have assessed the overall QoL aspects using the generic and disease‐specific scales.

In the selected literature for the review, the majority of the study populations were based on oro‐pharyngeal cancer patients. The QoL of such patients is a crucial aspect. Due to the nature of the lesions and the management modalities including the surgical approach, there is a higher chance of compromising vital functions such as speech, swallowing, speech, taste sensation, and smell. Therefore, following surgical excision of the lesions, QoL may be affected specially in relation to the physical, psychological, social, and emotional well‐being.^27^ QoL/HRQOL is increasingly being utilized in clinical studies, frequently to assess the outcome of an intervention or a therapy in complementary to traditional endpoints.^28^ It was interesting to note that four studies included in the review were randomized controlled trials. In clinical trials, the inclusion of QoL will also allow the clinician to compare different treatment modalities thereby assessing the benefits of the applied medical management. This scoping review demonstrates the beneficial effects on the QoL of dental patients treated with a laser‐assisted surgical procedure. Thus, where the facilities are available the clinicians could use this information to decide the mode of approach in terms of surgical technique. The review also suggests the need to assess the extension of such lesions in determining the excision technique.

According to the systematic review by Pequeno et al., the QoL instruments could assist the clinician in making informed decisions on the disease management.^10^ Therefore, these findings may also be used by clinicians and decision‐makers to determine the ways to efficiently prioritize and maximize the use of available healthcare system resources.

### 4.2 Strengths of the study

This scoping review covered a wider range of databases, and the initial retrieved articles were analyzed through a blind process by two investigators. Also, the selection of the research articles was done through strict eligibility criteria. This review included findings from four randomized controlled trials, which are considered as the gold standard for assessing the efficacy of a treatment. Some studies had longer follow‐up periods which could reflect a better scope of the postsurgical experience and perception of the patient.^16^, ^20^, ^21^, ^22^, ^23^

### 4.3 Limitations of the study

The included research had diverse settings in relation to the population characteristics, surgical technique, laser type, and heterogenicity of the assessed duration making it difficult to make conclusive findings. It is also important to note that such settings could impede comparisons between population groups with similar characteristics thereby compromising the statistical reliability and validity, thus the quality of the findings. Furthermore, due to the differences in the domains assessed in different QoL/HRQOL tools, direct comparison of the achieved scores is not possible. Moreover, the study methodologies were not formally assessed to evaluate the quality of the interventions. Most of the questionnaire data were gathered from self‐reported information which may have an implication on the outcome of QoL. The search was limited to articles in English language, with full‐text access. Therefore, similar articles that may have been published in languages other than English and articles without full access were not evaluated.

## 5 CONCLUSION

This scoping review provides a comprehensive overview of the current literature on the QoL of dental patients treated with laser surgical techniques. Although the studies have considered the outcomes in different time intervals, the overall results suggest that laser surgery is an effective and safe treatment option and can result in improved patients' QoL. Nevertheless, further studies are needed to corroborate the findings of this review, particularly in terms of generalizability, outcomes defined in specific time scales and the long‐term consequences of laser surgery on patients' QoL. More contextual and longitudinal qualitative research will be required to further assess these aspects. Future research would also be beneficial to focus on the preferences for suitable tools to assess the QoL.

## AUTHOR CONTRIBUTIONS

**Dhanushka Leuke Bandara**: Formal analysis; investigation; resources; software; validation; visualization; writing—original draft; writing—review and editing. **Kehinde K. Kanmodi**: Conceptualization; data curation; formal analysis; funding acquisition; investigation; methodology; project administration; resources; software; supervision; validation; visualization; writing—original draft; writing—review and editing. **Afeez A. Salami**: Data curation; investigation; methodology; resources; software. **Jimoh Amzat**: Writing—review and editing. **Ruwan D. Jayasinghe**: Formal analysis; investigation; project administration; resources; supervision; validation; writing—original draft; writing—review and editing.

## CONFLICT OF INTEREST STATEMENT

Kehinde Kazeem Kanmodi is an Editorial Board member of Health Science Reports and a co‐author of this article. To minimize bias, they were excluded from all editorial decision‐making related to the acceptance of this article for publication. The remaining authors declare no conflict of interest.

## ETHICS STATEMENT

Ethical clearance was not applicable for this study, as the study did not collect data from human or animal subjects but an open research repository. Furthermore, obtaining the informed consent was not applicable since the study data were based on anonymous secondary data that have been reported in the existing literature.

## TRANSPARENCY STATEMENT

The corresponding author, Kehinde Kazeem Kanmodi, affirms that this manuscript is an honest, accurate, and transparent account of the study being reported; that no important aspects of the study have been omitted; and that any discrepancies from the study as planned (and, if relevant, registered) have been explained.

## Data Availability

Data sharing is not applicable to this article as no new data were created or analyzed in this study.

## APPENDIX A

### A.1

See Table A4.

**Table A1:** Search string for PubMed database search.

Tag	Subject search	Search string
#1	Laser	laser[Title/Abstract]
#2	Dental surgery	(((dental surgery[Title/Abstract]) OR (oral surgery[Title/Abstract])) OR (maxillofacial surgery[Title/Abstract])) OR (periodontal surgery[Title/Abstract])
#3	Quality of life	(Quality of life[Title/Abstract]) OR (wellbeing[Title/Abstract])
#4	#1 AND #2 AND #3	(#1) AND (#2) AND (#3)

**Table A2:** Search string for SCOPUS database search.

Tag	Subject search	Search string
#1	Laser	TITLE‐ABS‐KEY (laser)
#2	Dental surgery	(TITLE‐ABS‐KEY (dental AND surgery) OR TITLE‐ABS‐KEY (oral AND surgery) OR TITLE‐ABS‐KEY (maxillofacial AND surgery) OR TITLE‐ABS‐KEY (periodontal AND surgery))
#3	Quality of life	(TITLE‐ABS‐KEY (quality AND of AND life) OR TITLE‐ABS‐KEY (wellbeing))
#4	#1 AND #2 AND #3	(#1) AND (#2) AND (#3)

**Table A3:** Search string for other database (APA PsycInfo and CINAHL Complete) search via EBSCO interface.

Tag	Subject search	Search string
S1	Laser	AB laser
S2	Dental surgery	AB dental surgery OR AB oral surgery OR AB maxillofacial surgery OR AB periodontal surgery
S3	Quality of life	AB quality of life OR AB wellbeing
S4	S1 AND S2 AND S3	S1 AND S2 AND S3

**Table A4:** Publications included for full‐text screening and their screening outcome.

S/N	Citations	Screening outcome	
		**Include**	**Exclude**
1	Parimbelli E, Simon C, Soldati F, Duchoud L, Armas GL, de Almeida JR, Quaglini S. Quality of life and health‐related utility after trans‐oral surgery for head and neck cancers. Health Qual Life Outcomes. 2021 Nov 3;19(1):250. doi:10.1186/s12955-021-01836-3. PMID: 34732202; PMCID: PMC8565022.		Excluded (Wrong study population)
2	Liu Y, Peng Q, Liu B, Wang Z, Cao Q. Er, Cr:YSGG Laser Therapy for Drug‐Induced Gingival Overgrowth: A Report of Two Case Series. Front Surg. 2022 May 24;9:922649. doi:10.3389/fsurg.2022.922649. PMID: 35686211; PMCID: PMC9171107.		Excluded (Wrong study design)
3	Gardenal N, Rigo S, Boscolo Nata F, Fernández‐Fernández MM, Boscolo‐Rizzo P, Gatto A, Tirelli G. The CO_2_ waveguide laser with flexible fiber in transoral resection of oral and oropharyngeal cancers: a retrospective cohort study on postoperative and quality of life outcomes. Lasers Med Sci. 2022 Apr;37(3):1755‐1762. doi:10.1007/s10103-021-03430-x. Epub 2021 Sep 30. PMID: 34591217.	Yes	
4	Rosenthal M, Baser RE, Migliacci J, Boyle JO, Morris LGT, Cohen MA, Singh B, Shah JP, Wong RJ, Patel S, Ganly I. Flexible fiber‐based CO_2_ laser vs monopolar cautery for resection of oral cavity lesions: A single center randomized controlled trial assessing pain and quality of life following surgery. Laryngoscope Investig Otolaryngol. 2021 Jul 10;6(4):690‐698. doi:10.1002/lio2.572. PMID: 34401493; PMCID: PMC8356859.	Yes	
5	Bußmann L, Laban S, Wittekindt C, Stromberger C, Tribius S, Möckelmann N, Böttcher A, Betz CS, Klussmann JP, Budach V, Muenscher A, Busch CJ. Comparative effectiveness trial of transoral head and neck surgery followed by adjuvant radio(chemo)therapy versus primary radiochemotherapy for oropharyngeal cancer (TopROC). BMC Cancer. 2020 Jul 29;20(1):701. doi:10.1186/s12885-020-07127-2. PMID: 32727416; PMCID: PMC7389683.		Excluded (Wrong publication type)
6	Forounghiasl P. Cautery versus laser excision of oral mucocoele. J paed Surg case reports. 2019; 47(4):101251. doi:10.1016/j.epsc.2019.101251		Excluded (wrong publication type)
7	Breeze J, Rennie A, Dawson D, Tipper J, Rehman KU, Grew N, Pigadas N. Patient‐reported quality of life outcomes following treatment for oral cancer. Int J Oral Maxillofac Surg. 2018 Mar;47(3):296‐301. doi:10.1016/j.ijom.2017.09.001. Epub 2017 Sep 22. PMID: 28943022.	Yes	
8	Ozcelik O, Seydaoglu G, Haytac CM. Diode laser for harvesting de‐epithelialized palatal graft in the treatment of gingival recession defects: a randomized clinical trial. J Clin Periodontol. 2016 Jan;43(1):63‐71. doi:10.1111/jcpe.12487. Epub 2016 Jan 21. PMID: 26660000.	Yes	
9	Broccoletti R, Cafaro A, Gambino A, Romagnoli E, Arduino PG. Er:YAG Laser Versus Cold Knife Excision in the Treatment of Non‐dysplastic Oral Lesions: A Randomized Comparative Study for the Postoperative Period. Photomed Laser Surg. 2015 Dec;33(12):604‐9. doi.org/10.1089/pho.2015.3967. Epub 2015 Nov 20. PMID: 26588688.	Yes	
10	Giovannacci I, Mergoni G, Meleti M, Merigo E, Fornaini C, Manfredi M, Bonanini M, Vescovi P. Postoperative discomfort in oral soft tissue surgery: a comparative perspective evaluation of Nd:YAG Laser, quantic molecular resonance scalpel and cold blade. Minerva Stomatol. 2015 Feb;64(1):9‐20. English, Italian. PMID: 25660590.	Yes	
11	Luna‐Ortiz K, Campos‐Ramos E, Pasche P, Mosqueda‐Taylor A. Oral mucosal melanoma: conservative treatment including laser surgery. Med Oral Patol Oral Cir Bucal. 2011 May 1;16(3):e381‐5. doi.org/10.4317/medoral.16.e381. PMID: 20711113.		Excluded (wrong publication type)
12	Zuydam AC, Lowe D, Brown JS, Vaughan ED, Rogers SN. Predictors of speech and swallowing function following primary surgery for oral and oropharyngeal cancer. Clin Otolaryngol. 2005 Oct;30(5):428‐37. doi.org/10.1111/j.1365-2273.2005.01061.x. PMID: 16232247.	Yes	
13	Rogers SN, Lowe D, Fisher SE, Brown JS, Vaughan ED. Health‐related quality of life and clinical function after primary surgery for oral cancer. Br J Oral Maxillofac Surg. 2002 Feb;40(1):11‐8. doi:10.1054/bjom.2001.0706. PMID: 11883963.	Yes	
14	Rogers SN, Lowe D, Brown JS, Vaughan ED. The relationship between length of stay and health‐related quality of life in patients treated by primary surgery for oral and oropharyngeal cancer. Int J Oral Maxillofac Surg. 2001 Jun;30(3):209‐15. doi:10.1054/ijom.2001.0040. PMID: 11420903.	Yes	
15	Rogers SN, Lowe D, Brown JS, Vaughan ED. The University of Washington head and neck cancer measure as a predictor of outcome following primary surgery for oral cancer. Head Neck. 1999 Aug;21(5):394‐401. 10.1002/(sici)1097-0347(199908)21:5<394::aid-hed3>3.0.co;2-q. PMID: 10402518.	Yes	
